# A Locus Identified on Chromosome18P11.31 is Associated with Hippocampal Abnormalities in a Family with Mesial Temporal Lobe Epilepsy

**DOI:** 10.3389/fneur.2012.00124

**Published:** 2012-08-10

**Authors:** Cláudia V. Maurer-Morelli, Rodrigo Secolin, Márcia E. Morita, Romênia R. Domingues, Rafael B. Marchesini, Neide F. Santos, Eliane Kobayashi, Fernando Cendes, Iscia Lopes-Cendes

**Affiliations:** ^1^Department of Medical Genetics, Faculty of Medical Sciences, University of CampinasCampinas, São Paulo, Brazil; ^2^Department of Neurology, Faculty of Medical Sciences, University of CampinasCampinas, São Paulo, Brazil

**Keywords:** genetics, microsatellites, linkage study, 18p11.31, hippocampal abnormalities

## Abstract

We aimed to identify the region harboring a putative candidate gene associated with hippocampal abnormalities (HAb) in a family with mesial temporal lobe epilepsy (MTLE). Genome-wide scan was performed in one large kindred with MTLE using a total of 332 microsatellite markers at ∼12 cM intervals. An additional 13 markers were genotyped in the candidate region. Phenotypic classes were defined according to the presence of hippocampal atrophy and/or hyperintense hippocampal T2 signal detected on magnetic resonance imaging. We identified a significant positive LOD score on chromosome 18p11.31 with a *Z*_max_ of 3.12 at D18S452. Multipoint LOD scores and haplotype analyses localized the candidate locus within a 6-cM interval flanked by D18S976 and D18S967. We present here evidence that HAb, which were previously related mainly to environmental risk factors, may be influenced by genetic predisposition. This finding may have major impact in the study of the mechanisms underlying abnormalities in mesial temporal lobe structures and their relationship with MTLE.

## Introduction

The association between mesial temporal lobe epilepsy (MTLE) and hippocampal sclerosis (HS) has been well established; as well as the use of hippocampal atrophy (HA) and abnormal hippocampal signal, observed on magnetic resonance imaging (MRI), as an *in vivo* surrogate marker of HS (Berkovic et al., 1991; Cendes et al., 1993; Van Paesschen et al., 1997). Although the precise pathogenesis of mesial temporal sclerosis (MTS) and its relationship with MTLE is not completely clarified, it has been frequently associated with environmental predisposing factors, mainly an increased incidence of prolonged childhood febrile seizures (Abou-Khalil et al., 1993; VanLandingham et al., 1998).

We have described previously clinical and imaging features of a type of MTLE associated with HA, showing familial recurrence and low frequency of febrile seizures (Kobayashi et al., 2001, 2002, 2003). Most affected individuals in familial MTLE have a mild phenotype with a benign form of MTLE (Kobayashi et al., 2001). HA with or without hyperintense T2 signal on MRI was observed in most patients, including those with a single focal seizure, seizure remission (Kobayashi et al., 2003) as well as in 34% of asymptomatic first-degree relatives of patients with familial MTLE (Kobayashi et al., 2002). These reports suggest that hippocampal abnormalities (HAb) identified by MRI in familial MTLE have a genetic predisposition and it is not necessarily associated to seizures in all patients (Kobayashi et al., 2001; Coan et al., 2004).

In the present study we report the results of a genome-wide linkage study, which identifies the candidate region harboring a putative gene associated with MRI signs of HAb in a family with MTLE.

## Materials and Methods

### Family

Familial MTLE was identified when two or more individuals presented the diagnosis of MTLE, which was based on clinical and electroencephalographic (EEG) findings as defined by the International League against Epilepsy (ILAE) criteria (ILAE, 1989). For this study we selected one single informative kindred for linkage analysis, named F-10, in order to avoid the risk of genetic heterogeneity.

### Magnetic resonance imaging

Magnetic resonance imagings were performed in a 2-T scanner including thin (1–3 mm) coronal T_1_-weighted images and T_2_-weighted coronal images, perpendicular to long axis of the hippocampal formation as described in Cendes et al. (1998) and Kobayashi et al. (2001, 2002).

Manual volumetry was performed using software developed by the National Institutes of Health (NIH-Image, National Institutes of Health, Bethesda, MD, USA) and anatomic guidelines were based on a standard protocol. Hippocampal volumes and asymmetry index (AI) for each patient (defined as the ratio of the smaller by the larger hippocampus) were compared to 30 healthy controls. Values that were 2 SDs below the mean values of control group were considered abnormal. Visual analysis was also performed looking for increased T2 signal and abnormal shape and axis of the hippocampus. Presence of HAb was identified if any abnormality was present.

Prior to genetic analysis, family members were classified into three phenotypic classes according to MRI findings: (1) *affected*: individuals with HAb detected by MRI, defined as: (i) HA, (ii) hyperintense T2 signal, (iii) abnormal shape or axis of the hippocampus, or (iv) any combination of these three findings (Kobayashi et al., 2002); (2) *unaffected*: individuals with no HAb detected by MRI; (3) *unknown*: individuals with no MRI information. Previous to enrollment, all family members provided informed consent which was approved by the Research Ethics Committee of our Institution.

### Genotyping

DNA was isolated from peripheral lymphocytes of fresh blood by standard methods (Sambrook et al., 1989) and genotyped for an in-house customized panel of 332 microsatellite markers regularly spaced at ∼12 cM intervals throughout the 22 autosomal chromosomes. Microsatellite markers were chosen from the sex-average map of the Marshfield Human Genetic Map^1^ with a polymorphism information content (PIC) >75%. The correct orientation of markers was obtained using the NCBI Map Viewer^2^. Genotyping was carried out using capillary electrophoresis on MegaBACE^™^ 1000 96-capillary sequencers (GE Healthcare, Buckinghamshire, UK). The fluorescent dye-labeled primers (FAM^™^, VIC^™^, NED^™^) were customized or chosen from the MD10 panel set (Applied Biosystems, Foster City, CA, USA). Samples amplified by PCR and labeled with different dyes were pooled in a final volume of 40 μl H_2_O (1:20 for FAM^™^, 1:20 for VIC^™^, and 1:10 for NED^™^) in a 96-wells plate. Two microliters were transferred to a MegaBACE 96-wells plate and mixed with 8 μl of a loading solution (for each reaction: 7.75 μl of 0.1% Tween 20 in H_2_O + 0.25 μl of internal size standard labeled with ROX^™^ dye, ET-550R; GE Healthcare, Buckinghamshire, UK). Automatic calling of genotypes was based on the Fragment Profiler Software v1.2 (GE Healthcare, Buckinghamshire, UK). In addition we used a software developed in-house for processing output data from Fragment Profiler and to estimate allelic frequency (Secolin et al., 2008). Mendelian inconsistencies were evaluated by PEDCHECK program (O’Connell and Weeks, 1998).

### Fine mapping

Thirteen additional markers were genotyped on chromosome 18p in order to refine the candidate region: telomere, D18S476, D18S1098, D18S481, D18S1154, D18S52, D18S1132, D18S976, D18S1376, D18S967, D18S1163, D18S464, D18S1150, and D18S1158, centromere.

### Linkage analysis

Linkage analysis was performed under the assumption of a dominant mode of inheritance (Secolin et al., 2010) with 85% penetrance which was calculated based on data obtained directly from F-10 using a method proposed by Wang et al. (2006). Gene frequency of the mutated allele was set at 0.00025. Two-point LOD scores were calculated using the MLINK program, version 5.2 (CEPH, University of Utah and Columbia University, 1990), from the LINKAGE computer package (Lathrop and Lalouel, 1984; Terwilliger and Ott, 1994). In order to further narrow down the candidate region on chromosome 18p we carried out a multipoint linkage analysis using the LINKMAP support program from the LINKAGE package (Lathrop et al., 1985).

## Results

**Figure 1:**
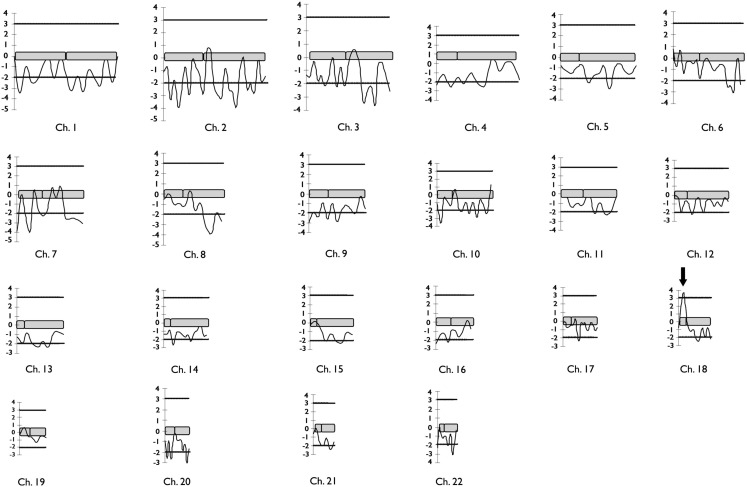
**Diagram representing the results of two-point LOD scores for the 332 microsatellites markers genotyped in the genome-wide linkage study of F-10 segregating familial mesial temporal lobe epilepsy**. Horizontal lines mark the upper and lower limits for LOD score significance. The arrow indicates the single significant positive LOD score found in the genome scan.

A total of 28 individuals, including 14 patients, were genotyped. Clinical and MRI data of F-10 are shown in Table 2. The results of the genome-wide linkage study are summarized in Figure 1. Overall, we found only one single significant positive LOD score on chromosome 18p (Figure 1). Fine mapping of the candidate region identified a Zmax of 3.12 at þeta = 0.0 at the D18S452 locus (Table 1).

**Table 1:** **Two-point LOD scores for the markers genotyped in the candidate region for familial temporal lobe epilepsy in the family F-10**.

Marker	Recombination fraction (θ)								
	0.00	0.05	0.10	0.15	0.20	0.25	0.30	0.35	0.40
**F-10**									
D18S476	2.36	2.33	2.20	2.00	1.77	1.50	1.20	0.88	0.54
D18S1098	1.17	1.23	1.20	1.11	0.99	0.84	0.66	0.47	0.26
D18S481	2.17	2.40	2.40	2.29	2.09	1.82	1.51	1.14	0.73
D18S1154	1.37	1.45	1.43	1.35	1.23	1.09	0.92	0.73	0.51
D18S52	2.21	2.40	2.37	2.24	2.04	1.77	1.46	1.10	0.70
D18S1132	1.82	2.07	2.10	2.00	1.83	1.58	1.29	0.95	0.58
D18S976	1.87	2.13	2.17	2.08	1.91	1.67	1.39	1.06	0.70
D18S1376	0.50	0.46	0.42	0.38	0.33	0.28	0.23	0.18	0.12
D18S452	3.12	3.10	2.95	2.72	2.43	2.09	1.70	1.28	0.83
D18S967	−0.00	−0.00	−0.00	−0.00	0.00	0.00	0.00	0.00	0.00
D18S1163	−0.83	−0.52	−0.34	−0.23	−0.15	−0.09	−0.06	−0.03	−0.01
D18S464	−0.03	0.01	0.04	0.05	0.06	0.07	0.06	0.06	0.04
D18S1150	−3.97	−2.58	−1.72	−1.13	−0.71	−0.39	−0.17	−0.03	0.04
D18S1158	−2.95	−1.56	−0.99	−0.63	−0.40	−0.24	−0.13	−0.07	−0.03

**Figure 2:**
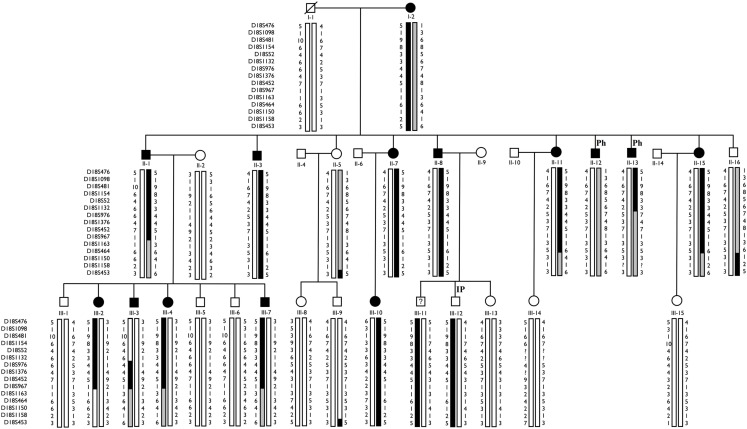
**Haplotype analysis of F-10 for 14 markers localized on chromosome 18p**. The haplotype inherited by affected individuals is represented by blackened vertical bars. Family members who did not participate in the linkage study are not shown. Individual III-3 shows a critical recombination event. IP, Incomplete Penetrance; Ph, Phenocopy.

Haplotype analysis showed 12 affected individuals segregating the haplotype predicted to carry the candidate gene in F-10, indicating a critical region of 6 cM between markers at D18S976 and D18S967 loci (see individual III-3 in Figure 2).

**Figure 3:**
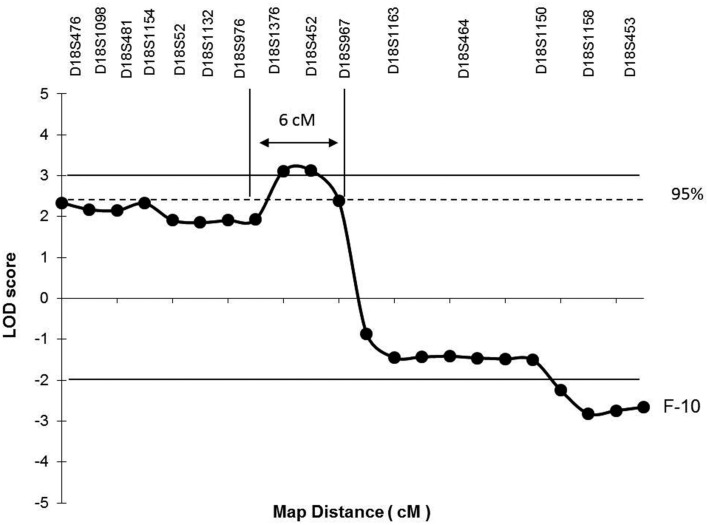
**Multipoint linkage analysis on chromosome 18p depicting the candidate region for the putative gene causing the phenotype in F-10**. Horizontal lines mark the upper and lower limits for LOD score significance. The single horizontal dotted line indicates the upper limit of the 95% confidence interval calculated for the maximum LOD score achieved. Arrows between vertical lines identify the critical region (6 cM) expected to contain the candidate gene flanked by markers at D18S967 and D18S976 loci.

However, the “affected” haplotype was also present in two individuals that are obligatory carries of the candidate gene mutation (individuals III-11 and III-12), but who were phenotypically classified as unknown and unaffected, respectively. In addition, haplotype analysis also showed two individuals who were considered affected, but who did not carry the “affected” haplotype (individuals II-12 and II-13). Multipoint analysis (Figure 3) confirms the mapping of the candidate region to a ∼6-cM interval between loci D18S976 and D18S967.

## Discussion

We identified a candidate locus on chromosome 18p11.31 associated with HAb in a family with MTLE.

Over the past 20 years, the progress in molecular genetics has lead to the identification of candidate loci and mutated genes in many epilepsy syndromes (Ottman, 2001). In temporal lobe epilepsy (TLE), a digenic inheritance with loci on 1q25–31 and 18qter and another locus on 12q22–q23.3 were described for familial TLE with febrile seizures (Baulac et al., 2001; Claes et al., 2004). Subsequently, a locus for familial MTLE was identified on chromosome 4q13.2–q21.3 by Hedera et al. (2007), but in contrast with F-10, none of the patients reported had MRI signs of HAb. Therefore, it becomes clear that locus heterogeneity is present even within the so-called familial forms of MTLE confirming the trend already seen in several other types of familial epilepsy syndromes (Baulac et al., 2001; Claes et al., 2004; Hedera et al., 2007).

Here we report a family which is part of a larger cohort showing a distinct type of familial MTLE associated with MRI signs of HAb, which also presents a low frequency of febrile seizures (Kobayashi et al., 2001). In the present study we used MRI signs of HAb to classify individuals as affected, independently of the presence of seizures (individual I-2, F-10; Kobayashi et al., 2002, 2003; Coan et al., 2004).

Although we had a reliable biological maker for phenotypic characterization, there were still difficulties with the MRI characterization of all individuals enrolled in the study. We could not ascertained the MRI-phenotypic status of individual III-11, since he presented claustrophobia and was unable to complete the exam; therefore, individual III-11 was considered unknown despite the fact that he carries the affected haplotype; thus, at this point we cannot completely exclude the possibility that he may have an abnormal MRI. Individual III-12 carries the affected haplotype but he had no MRI abnormalities, even when he was subsequently examined in a 3T MRI scan (data not shown); therefore, he may represent an example of incomplete penetrance. In fact, we estimated the penetrance in our linkage analysis at 85%. Incomplete penetrance is not unusual in familial epilepsies (Johnson et al., 1996; Striano et al., 2008) and it accounts for part of the complex relationship observed between genotype and phenotype in different epilepsy syndromes (Anderson et al., 2002; Ottman, 2005).

In addition, we identified two individuals (II-12 and II-13), who were considered affected, since they had MRI abnormalities (Table 2) but did not present the “affected” haplotype (Figure 2). Individual II-12 had an increased T2 signal and abnormal shape of the hippocampus and individual II-13 had left HA. Individual II-12 has a history of heavy alcohol intake, a well-known cytotoxic agent which can cause hippocampal damage (Sullivan et al., 1995; Agartz et al., 1999); thus, we raise the possibility that one or both individuals could be phenocopies. Therefore, at this point, we cannot completely exclude the possibility that the abnormalities seen on MRI of both individuals may be due to factors other than the mutated gene on chromosome 18p11.31 segregating in this family.

**Table 2:** **Clinical information for family F-10**.

Individual	Clinical group	Volume	Increased T2 signal	Abnormal shape or axis	Seizure onset	EEG	Seizures	“Affected” haplotype
I-2	asymp	BHA	Yes	Yes	–	NA	–	Yes
II-1	MTLE remis	normal	No	Yes	9	NA	SP, SG	Yes
II-3	single sz	LHA	No	No	35	NA	SP, CP	Yes
II-5	G	Normal	No	No	12	Normal	G	No
II-7	bg MTLE	Normal	No	Yes	Childhood	NA	CP, SG	Yes
II-8*	G	BHA	Yes	Yes	Childhood	NA	G	Yes
II-11	MTLE remis	RHA	No	Yes	19	NA	SP, CP, SG	Yes
II-12**	MTLE remis	Normal	Yes	Yes	8	NA	SP, G	No
II-13	MTLE remis	LHA	Yes	Yes	8	NA	SP, CP	No
II-15	bg MTLE	Normal	No	Yes	6	NA	SP, CP, SG	Yes
II-16	asymp	Normal	No	No	–	NA	–	No
III-1	asymp	NA	NA	NA	–	NA	–	No
III-2	bg MTLE	LHA	Yes	Yes	2	NA	SP, CP, SG	Yes
III-3	bg MTLE	BHA	No	Yes	5	Normal	SP, CP, SG	Yes
III-4	bg MTLE	BHA	Yes	Yes	14	LT ED	SP, CP, SG	Yes
III-5*	G	Normal	No	No	14	RT SW	G	No
III-6	asymp	Normal	No	No	–	NA	–	No
III-7	bg MTLE	Normal	No	Yes	6	RT SW	CP, SG	Yes
III-8	asymp	NA	NA	NA	–	NA	–	No
III-9	asymp	NA	NA	NA	–	NA	–	No
III-10	G	BHA	Yes	Yes	5	NA	G	Yes
III-11	single sz	NA	NA	NA	19	NA	SP, SG	Yes
III-12	asymp	No	No	No	–	NA	–	Yes
III-13***	asymp	Normal	No	No	–	NA	–	No
III-14	asymp	Normal	No	No	–	NA	–	No
III-15	SP	Normal	No	No	8	Normal	SP	No

LHA, left hippocampal atrophy; RHA, right hippocampal atrophy; BHA, bilateral hippocampal atrophy; SP, simple partial seizure; bg MTLE, benign mesial temporal lobe epilepsy; MTLE remis, mesial temporal lobe epilepsy with remission; G, generalized; asymp, asymptomatic; single sz, single seizure; RT SW, right temporal slow waves; LT ED, left temporal epileptiform discharges; CP, complex partial seizures; SG, secondary generalized seizures; G, generalized. NA, not available. *Nocturnal unwitnessed generalized seizures, impossible to define if the onset was partial; **G seizures from 8 to 12 years; ***left frontal glial tumor.

**Figure 4:**
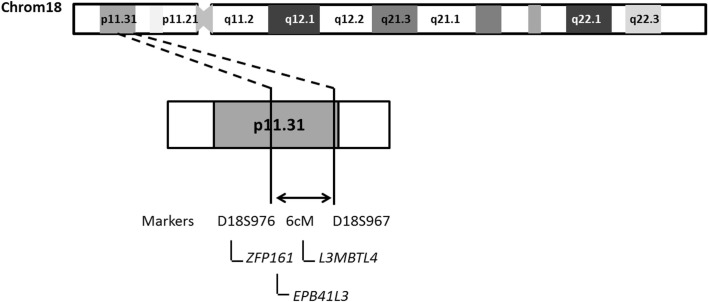
**Schematic representation of chromosome 18 indicating the region likely to contain the candidate locus identified in F-10**.

The combined results of multipoint LOD scores and haplotype data points to a candidate interval of 6 cM between D18S976 and D18S 967. A search in the NCBI^3^ and Ensembl database^4^ showed at least three genes (Figure 4), which could play a role in the pathogenesis of HS such as: *ZFP161* (*zinc finger protein, 161*, MIM 602126) a gene involved in CNS development and recently pointed out as a candidate for holoprosencephaly (Sobek-Klocke et al., 1997; Lee et al., 2004); *L3MBTL4* [*L(3)mbt-like 4 (Drosophila)*, gene ID91133], which functions as a cell adhesion molecule and *EPB41L3* (*erythrocyte membrane protein band 4.1-like3*, MIM 605331) that encodes a family of proteins likely to take part in skeletal structures of cells and it is highly expressed in the brain (Peters et al., 1998). In addition, we identified the *TTMA gene* (two transmembrane domain family member A, gene ID 645369), and five hypothetical proteins localized in this candidate region. Mutation screening in candidate genes located within the 18p11.31 region is under way in order to identify the gene responsible for this disease.

In conclusion, we identified a candidate locus for HAb associated with familial MTLE on chromosome 18p11.31 suggesting that a major gene predisposing to HS is present in patients from family F-10. This is strong evidence suggesting that genetic causes leading to hippocampal damage can also be related to MTLE.

## Conflict of Interest Statement

The authors declare that the research was conducted in the absence of any commercial or financial relationships that could be construed as a potential conflict of interest.

## Footnotes

^1^http://www.research.marshfieldclinic.org/genetics

^2^http://www.ncbi.nlm.nih.gov/mapview

^3^http://www.ncbi.nlm.nih.gov

^4^http://www.ensembl.org

## Statement

We confirm that we have read the Journal’s position on issues involved in ethical publication and affirm that this report is consistent with those guidelines.
